# Epidermal growth factor receptor tyrosine kinase inhibitors

**DOI:** 10.1038/sj.bjc.6601873

**Published:** 2004-05-11

**Authors:** M Ranson

**Affiliations:** 1 Department of Medical Oncology, University of Manchester, Christie Hospital NHS Trust, Wilmslow Road, Manchester M20 4BX, UK

**Keywords:** epidermal growth factor receptor, tyrosine kinase, gefitinib, erlotinib, PKI-166, GW-572016, EKB-569, CI-1033, ZD6474

## Abstract

Activation of the epidermal growth factor receptor (EGFR) has been linked to tumour proliferation, invasion, metastasis and angiogenesis in epithelial tumours. Inhibitors of the EGFR have emerged as promising anticancer agents and two main approaches have been developed, humanised monoclonal antibodies and tyrosine kinase inhibitors. This review discusses the current status of EGFR tyrosine kinase inhibitors (EGFR-TKIs) that have entered clinical development. EGFR-TKIs are generally well tolerated and can sometimes produce impressive tumour regression in patients with advanced non-small-cell lung cancer. However, highly predictive or surrogate markers of activity have not been identified and there remains a need for translational research in their future development.

Trans-membrane receptor tyrosine kinases play an important role in the modulation of growth factor signalling. The epidermal growth factor receptor (EGFR) is a member of a family of four closely related receptors: EGFR (or erbB1), HER2/neu (erbB2), HER3 (erbB3) and HER4 (erbB4). The EGFR mediates the actions of multiple ligands including epidermal growth factor, transforming growth factor-*α*, amphiregulin and heparin-binding EGF, and may also be constitutively activated by mutation. EGFR signalling has been reported to be important for tumour cell proliferation, inhibition of apoptosis, angiogenesis, metastasis and sensitivity to chemotherapy and radiotherapy (Ritter and Arteaga, 2003; Arteaga, 2003a). EGFR signalling is also important in normal epithelial cell biology and thus defining a therapeutic window for EGFR inhibitors has been an important goal in their clinical evaluation. EGFR signalling is complex. Homodimerisation and heterodimerisation with other EGFR family members is known to occur (Jorissen *et al*, 2003). Within the EGFR family, HER2 appears to be a preferred heterodimer partner.

The quinazolines, gefitinib and erlotinib, which are the most advanced in clinical development, are competitive inhibitors at the tyrosine kinase ATP binding site. Irreversible inhibitors that bind to specific cysteines in the ATP-binding pocket of EGFR family receptors have been developed. Examples include CI-1033 and EKB-569. The structural homology between EGFR receptor members has also been exploited for the development of inhibitors that block multiple members of the EGFR family (Table 1

**Table 1:** EGFR TKIs in clinical development

**Drug**	**Other names**	**Description**	**Status**
Gefitinib	ZD1839, Iressa	Quinazoline, reversible, EGFR TKI	Phase II/III, launched Japan, Australia, USA
Erlotinib	OSI-774, Tarceva	Quinazoline, reversible, EGFR TKI	Phase II/III
PKI-166	CGP59326	Pyrrolo-pyrimidine, reversible, dual EGFR/erbB2 TKI	Phase I/II
GW-572016	GW-2016	Thio-quinazoline, reversible, dual EGFR/erbB2 TKI	Phase I/II
Canertinib	CI-1033, PD-0183805	Pyrido-pyrimidine, irreversible, Pan-erbB TK1	Phase II
EKB-569		Cyanoquinoline, irreversible, dual EGFR/erbB2 TKI	Phase I/II
ZD6474		Quinazoline, reversible, VEGFR/flk-1/KDR/EGFR TKI	Phase II

).

## PHARMACODYNAMICS

All the EGFR TKIs shown in Table 1 act at the ATP binding site in the catalytic domain of the receptor and *in vitro* inhibit EGFR tyrosine kinase at low nanomolar concentrations. A key finding is a lack of a close correlation between varying degrees of EGFR expression in tumour cells and their sensitivity to EGFR TKI inhibitors such that low-level EGFR tumours can be more sensitive than tumours with high receptor expression (Parra *et al*, 2002; Ciardiello *et al*, 2003).

Research in breast and other epithelial cell lines has suggested that HER-2 overexpressing tumours may be more sensitive to EGFR inhibitors such as gefitinib (Moasser *et al*, 2001). Others have reported that gefitinib can block HER-2 function by inducing the formation of inactive unphosphorylated EGFR/HER2 and EGFR/HER3 heterodimers and by blocking the formation of HER2/HER3 heterodimers (Anido *et al*, 2003). In cells that co-express HER2 and EGFR, ligand activation preferentially recruits HER2 into a heterodimeric complex that has increased stability and a different endocytic recycling, resulting in increased signalling potency (Lenferink *et al*, 1998). Such results suggest that EGFR TKIs should be tested in patients with EGFR+HER-2-positive breast cancer. However, it should be noted that the clinical activity of gefitinib has recently been reported to be unaffected by HER2 status in a small-scale trial in advanced non-small-cell lung cancer (NSCLC) (Cappuzzo *et al*, 2003).

The downstream molecular mechanisms underlying the antitumour actions of EGFR TKIs have been examined in preclinical models, with particular attention on mitogen-activated protein kinase (MAP kinase) and phosphatidylinositol 3′–kinase/Akt (PI3/Akt) pathways. In A431 cells, the antitumour effects of gefitinib are mediated via inhibition of both MAP kinase and PI3/Akt pathways resulting in apoptotic cell death. In NSCLC cell lines, however, more limited antiproliferative effects were accompanied by evidence of continued activity in one or both of these pathways (Janmaat *et al*, 2003). Early attempts to define mechanisms of resistance to TKIs suggests that the PI3/Akt pathway is important as a loss of PTEN, allowing high levels of Akt independent of EGFR control, blocks the effects of EGFR inhibitors, and persistent Akt activity is seen in resistant cells (Bianco *et al*, 2003; She *et al*, 2003).

Mechanistic information has also emerged from pharmacodynamic studies performed during early clinical trials. In a phase I/II pharmacodynamic trial in patients with metastatic colorectal cancer, gefitinib produced loss of immunohistochemical staining for activated EGFR, phosphorylated Akt and phosphorylated ERK in some but by no means all tumour biopsies (Daneshmand *et al*, 2003). Pharmacodynamic studies of skin biopsies before and during gefitinib therapy showed that doses well below the maximal tolerated dose (MTD) inhibited MAP kinase activation, increased p27 expression and reduced keratinocyte proliferation (Albanell *et al*, 2002). Using Erlotinib and monitoring EGFR signalling in skin biopsies, significant decreases in activated EGFR expression and increased expression of p27 as assessed by immunohistochemistry have been reported, but no significant effect was seen in phospho-ERK expression (Malik *et al*, 2003).

Attempts have been made to identify tumour phenotypic and genotypic profiles that confer sensitivity to EGFR TKIs (Perez-Soler *et al*, 2003). To identify genes that might be associated with sensitivity to gefitinib, a detailed expression profiling from a panel of human xenograft tumours has been undertaken (Zembutsu *et al*, 2003). An analysis of over 23 000 genes revealed 114 genes whose expression correlated significantly with sensitivity to gefitinib. These preclinical data need to be compared with preliminary data emerging from clinical trial tumour specimens (Natale *et al*, 2003).

## GEFITINIB AND ERLOTINIB

Gefitinib (ZD1839, Iressa) and erlotinib (OSI-774, Tarceva) are in advanced phase II/III development and other agents are in phase I/II trials Table 1. Gefitinib is currently licensed for use in relapsed advanced NSCLC in Japan, Australia and USA and applications are underway in other territories. Both agents have good oral bioavailability and are suitable for once daily administration. In phase I trials, skin rash and diarrhoea are dose limiting. Gefitinib appears to be active below its MTD. Evidence of therapeutic activity of gefitinib was seen at doses as low as 150 mg day^−1^ in patients with relapsed advanced NSCLC with anecdotal activity in other tumours (Baselga *et al*, 2002; Herbst *et al*, 2002; Ranson *et al*, 2002; Nakagawa *et al*, 2003). The MTD in these phase I trials was 600–1000 mg day^−1^. Multicentre phase II trials in patients with relapsed NSCLC after chemotherapy have confirmed that gefitinib and erlotinib have activity in this setting with partial response rates of around 15% (Perez-Soler *et al*, 2001; Kris *et al*, 2002; Fukuoka *et al*, 2003) (Table 2

**Table 2:** Summary of phase II trials of gefitinib and erlotinib monotherapy in relapsed advanced NSCLC

**Drug**	**Number of evaluable patients**	**Number of prior chemotherapy regimens**	**Dosing**	**Response rate (%)**	**Response+stable disease**	**Median survival^a^ (months)**	**Reference**
Gefitinib	209	1 or 2 (platinum, docetaxel containing)	250 mg day^−1^ *vs* 500 mg day^−1^	18.4	54.4	7.6	Fukuoka *et al* (2003)
IDEAL 1							
Gefitinib	216	At least 2 including *both* platinum and docetaxel	250 mg day^−1^ *vs* 500 mg day^−1^	11.8	42.2	6.5	Kris *et al* (2002)
IDEAL 2							
Erlotinib	56	At least 1 including platinum	150 mg day^−1^	12.3	39.0	8.5	Perez-Soler *et al* (2001)

a Survival from the start of EGFR TKI.

).

In a randomised trial of gefitinib 250 mg day^−1^ *vs* 500 mg day^−1^ in second or third line treatment of relapsed, advanced stage NSCLC (IDEAL 1 trial), the median survival of 210 randomised patients from the start of ZD1839 was 7.8 months with a median survival in responding patients of approximately 11 months (Fukuoka *et al*, 2003). In this study and a similar trial (IDEAL 2) in more heavily pretreated symptomatic NSCLC patients (Kris *et al*, 2002), about a third of patients reported improvement in lung cancer symptoms assessed by the FACT-L QOL instrument (Natale and Zaretsky, 2002). Very similar activity has been reported for erlotinib dosed at its MTD of 150 mg day^−1^ in a single arm phase II trial in 56 evaluable patients (Perez-Soler *et al*, 2001).

Erlotinib and gefitinib have been studied in phase II trials in patients with advanced squamous cell carcinoma of the head and neck (SCCHN). Response rates of approximately 5–10% have been reported with up to a third of patients showing stable disease (Cohen *et al*, 2003; Herbst, 2003). A phase II trial of gefitinib 500 mg day^−1^ in 52 patients with recurrent SCCHN showed a response rate of 10.6%. Survival in trials of EGFR TKIs in relapsed SCCHN are similar to those seen with other agents in this patient cohort, and randomised trials are now required.

Since antitumour activity has been seen below the MTD for gefitinib, defining an optimal dose of EGFR TKI may need to be based on an appraisal of other end points. The phase I trials of gefitinib were unusual in using expanded dosing cohorts so as to provide early opportunity for investigation of end points other than simply the MTD. Over 250 patients were enrolled in four Phase I trials of gefitinib, including 100 patients with relapsed advanced NSCLC (Baselga *et al*, 2002; Herbst *et al*, 2002; Ranson *et al*, 2002; Nakagawa *et al*, 2003). Partial responses in NSCLC were seen across a wide dose range (150–1000 mg day^−1^), and pharmacodynamic data showed that EGFR signalling was consistently inhibited in skin biopsies at all doses above 150 mg day^−1^ (Albanell *et al*, 2002). Dose levels of ⩾150 mg day^−1^ also achieved plasma levels, which were markedly inhibitory (above IC90 values) for most tumour cells *in vitro* (Ranson *et al*, 2002). Based upon this appraisal, two randomised phase II studies of gefitinib comparing 250 mg *vs* 500 mg day^−1^ in over 400 patients with relapsed NSCLC were conducted (Kris *et al*, 2002; Fukuoka *et al*, 2003) Both these trials showed greater toxicity (mainly rash and diarrhoea), with no additional therapeutic benefit from the higher dose. From these data, the conclusion can be drawn that for relapsed NSCLC, the optimal dose of gefitinib is 250 mg day^−1^, which is well below its MTD.

The approach taken with erlotinib has been quite different. Phase II and III studies have been conducted at the MTD dose of 150 mg day^−1^. Whether lower doses of erlotinib would be as effective as dosing at the MTD is currently unknown. Randomised trials with sufficient power to detect differences in efficacy would be needed to address this question. Analysis of data from a small study of 57 NSCLC patients treated with erlotinib in a phase II trial showed a significant positive correlation between skin rash and survival (Herbst, 2003), an observation that deserves to be examined in randomised dose comparator trials. Patients who are deriving therapeutic benefit are intrinsically more likely to have greater periods of drug exposure and thereby may have increased tendency to rash. To attempt to clarify whether dosing to MTD is required for erlotinib, a trial is being conducted to dose patients so as to achieve tolerable rash. It will be important to determine if this approach for erlotinib yields enhanced survival and clinical benefit.

Preclinical data have shown that the addition of EGFR TKIs (or anti-EGFR antibodies) enhances the activity of single agent cytotoxic drugs (reviewed Arteaga, 2003b). Such observations fuelled an expectation that the addition of gefitinib to cytotoxic chemotherapy regimens would result in improved efficacy. Randomised phase III trials involving over 2000 patients with stage III/IV NSCLC have been completed to test whether the addition of gefitinib to doublet chemotherapy improves survival. Two trials have been reported one with carboplatin/paclitaxel (INTACT 1), the other with cisplatin/gemcitabine (INTACT 2). Both studies clearly demonstrated that the addition of gefitinib (at either 250 or 500 mg day^−1^) did not improve the response rates, time to progression or survival compared to combination chemotherapy alone (Giaccone *et al*, 2004, Herbst *et al*, 2004). Indeed, historically, there has been no convincing evidence that triplet drug regimens are superior to optimised doublets in advanced NSCLC.

Randomised phase III trials of erlotinib in combination with carboplatin/paclitaxel (TRIBUTE TRIAL) or with cisplatin/gemcitabine (TALENT TRIAL) have completed patient recruitment, and in October 2003 OSI Pharmaceuticals announced that these trials were also negative and that the addition of erlotinib to these doublet chemotherapy regimens failed to produce a survival advantage over chemotherapy alone. An important unanswered question is whether either of these agents improves the efficacy of *single agent* chemotherapy. Phase II/III studies of gefitinib or erlotinib are underway in a wide range of solid tumours including head and neck cancer, prostate, breast, colorectal, ovarian, cervical, endometrial, pancreatic, glioblastoma and renal tumours and preliminary results have been reported in recent reviews (Herbst, 2003; Schiller, 2003).

The toxicity profile of gefitinib and erlotinib are remarkably similar; skin rash and diarrhoea being the most frequently encountered adverse effects. At current phase II/III dose levels, toxicity is usually grade 1 or 2 and rarely dose limiting. Diarrhoea usually responds well to antidiarrhoeal treatment; and it has been observed that skin toxicity can sometimes improve in the face of continued dosing (Ranson *et al*, 2002). The incidence of grade III/IV toxicity with gefitinib monotherapy was 8.7% in IDEAL 1 and 6.9% of patients in IDEAL 2. For monotherapy with erlotinib grade III/IV adverse events were reported in 30% of patients (Perez-Soler *et al*, 2001).

Shortly following the licensing of gefitinib in Japan in 2002, sporadic reports appeared of interstitial lung disease during gefitinib therapy (Ieki *et al*, 2003). The syndrome comprises an acute onset of bilateral pulmonary infiltrates accompanied by hypoxia. Some cases have proved fatal while others have improved following gefitinib withdrawal, steroids and supportive measures. The incidence in Japan has been reported at 1.7% (Inoue *et al*, 2003). This is higher than that reported worldwide of 1% in over 92 000 patients and of 0.38% in >39 000 patients as part of the compassionate use programme (Forsythe and Faulkner, 2003). Analysis of the data from the placebo controlled INTACT trials has been reported to show no difference in incidence of toxicities which could be included as interstitial lung disease. The mechanism of gefitinib-related interstitial lung disease is currently unclear.

## OTHER EGFR TKIS

CI-1033, PKI-166, EKB-569 and GW-572016, which produce inhibition of multiple EGFR family members, are in early phase clinical development. It will be important to clarify if these wider spectrum inhibitors have greater therapeutic potential than the specific EGFR1 TKIs.

### Canertinib (CI-1033, PD-0183805)

Canertinib, a water-soluble pyrido-pyrimidine under development by Pfizer, is a potent kinase inhibitor of all members of the erbB receptor family. Canertinib produces rapid, irreversible inhibition of EGFR kinase with an IC_50_ in the low nanomolar range and antitumour activity in EGFR and erbB2 dependent preclinical models (Slichenmyer *et al*, 2001). Like other EGFR TKIs it inhibits downstream signalling in both MAP kinase and PI3 kinase/Akt pathways. Phase I trials have been reported with canertinib using four different dosing schedules (Rinehart *et al*, 2002). On a 14-day schedule every 3 weeks, the MTD was 450 mg day^−1^ (Nemunaitis *et al*, 2003); the MTD was 250 mg day^−1^ on a 7-day on/7-day off schedule (Rowinsky *et al*, 2003), and 150 mg day^−1^ with continuous dosing (Rinehart *et al*, 2003). The drug is generally well tolerated, the most frequent adverse events being diarrhoea, skin rash and stomatitis (Rinehart *et al*, 2002; Nemunaitis *et al*, 2003; Rowinsky *et al*, 2003). At high intermittent dosing, hypersensitivity reactions were encountered. Phase II trials are in progress.

### PKI-166 (CGP59326)

This pyrrolo-pyrimidine, reversible, dual EGFR and erbB2 TKI is under clinical development by Novartis and entered clinical trials in 1999. PKI-166 has preclinical pharmacology typical of other class compounds, with inhibition of epithelial tumour growth and antiangiogenic actions (Baker *et al*, 2002), and the agent can potentiate the activity of gemcitabine in preclinical models of pancreatic cancer (Solorzano *et al*, 2001). In a phase I trial using a 2-week on/2-week off dosing schedule, dose-limiting skin rash and diarrhoea was encountered at 900 mg (Hoekstra *et al*, 2002). The drug was found to be very well tolerated when administered three times weekly (Murren *et al*, 2002).

### GW-572016 (GW-2016)

GW-572016 (GW-2016), a 6-thiazolylquinazoline, is a reversible, dual ErbB2 and EGFR TKI under development by GlaxoSmithKline. Phase I data have recently been reported with notable tumour responses seen in patients with trastuzumab refractory breast cancer and in NSCLC (Spector *et al*, 2003). Inhibition of phosphoAkt and inhibition of activated ERK1/2 in biopsies have been linked with tumour regression.

### EKB-569

EKB-569, a 3-cyanoquinoline, is an irreversible, dual inhibitor of EGFR and HER-2 tyrosine kinases. Phase I trials using both intermittent and continuous dosing have shown the drug to be generally well tolerated at doses up to the MTD of 75 mg day^−1^ (Hidalgo *et al*, 2002). Diarrhoea, skin rash, nausea, vomiting, stomatitis and anorexia are the most frequently reported adverse events. Phase II trials are currently underway.

### ZD6474

ZD6474 is an orally bioavailable VEGFR flk-1/KDR receptor (VEGFR-2) tyrosine kinase inhibitor of the quinazoline class. This compound has additional activity against EGFR and fms-like tyrosine kinase 4 (VEGFR3) (Ciardiello *et al*, 2003). Preclinical data suggest that ZD6474 may have extended activity compared to that seen with gefitinib (Matsumori *et al*, 2003). In keeping with its EGFR inhibitory actions, skin rash has been commonly encountered in phase I trials. Early phase II evaluation is in progress.

## EGFR TYROSINE KINASE INHIBITORS AND HORMONAL THERAPY

Evidence suggests that, at least in preclinical models, cross-talk between the oestrogen receptor (ER) and the EGF/HER2 receptor pathways is associated with endocrine therapy resistance (Nicholson *et al*, 2002). The treatment of wild-type MCF-7 breast cancer cells with tamoxifen and gefitinib has been reported to prevent development of tamoxifen resistance (Wakeling *et al*, 2001). The EGFR pathway may also play an important role in oestrogen-independent breast cancer, and one group has presented preclinical data suggesting that hormone independent cells are more sensitive to EGFR TKIs than wild-type cells (Gee *et al*, 2001). In xenografts of surgically removed DCIS breast tissue, gefitinib has been found to reduce proliferation and increase apoptosis, (Chan *et al*, 2000). The relevance of these models to clinical settings of hormone resistance and DCIS needs to be further tested.

## RADIOTHERAPY AND EGFR TYROSINE KINASE INHIBITORS

Tumour cell survival and repopulation following radiotherapy in epithelial tumors may be regulated by the activation and expression of EGFR and its ligands following radiation. Furthermore, the activation of downstream effectors of the EGFR signalling pathway have been shown to increase cellular resistance to ionizing radiation, suggesting that EGFR inhibitors may lead to a reduction in tumour cell repopulation and the modulation of cellular radiosensitivity (Harari and Huang, 2002). Additive to synergistic interactions have been observed with *in vitro* and *in vivo* studies of EGFR TKIs in combination with radiation; in some instances there is sequence dependence something that should be borne in mind in the clinical testing of these hypotheses.

## CONCLUSIONS

Identification of the clinical activity of erlotinib and gefitinib in NSCLC and SCCHN and the licensing of gefitinib for relapsed NSCLC in Japan, Australia and USA have been important recent developments in the field. New understanding of EGFR biology has also emerged from clinical trials. There appears to be no simple association between the level of EGFR1 expression and the clinical activity of EGFR TKIs; high EGFR expressing tumours do not constitute a group that is intrinsically more sensitive. The interplay between EGFR expression, receptor activation, ligand expression, levels of other EGFR members and downstream signalling proteins needs to be defined by further research. Unlike the paradigm of imatinib mesylate in gastrointestinal stromal tumours where patients exhibit a relatively homogeneous phenotype, there seems to be no easily identifiable human cancer phenotype with a strong EGFR dependence. The results from phase II trials of gefitinib in advanced recurrent NSCLC indicate that response seems to occur more frequently in patients with adenocarcinoma than with squamous carcinoma, but this observation requires confirmation with other EGFR inhibitors.

Preclinical and clinical research should help in identifying markers of EGFR TKI sensitivity and give pointers about mechanisms of resistance to EGFR TKIs. Given the complex interplay between EGFR family receptors it is not surprising that a simple relationship between EGFR expression and sensitivity is lacking. Evaluating downstream signalling components is more likely to be helpful in identifying patients likely to benefit from EGFR TKIs. Defining the mechanisms of resistance to EGFR inhibitors coupled with identifying the clinical and molecular profile of responding *vs* nonresponding patients in ongoing trials remains an important priority and should hopefully enable a more focused use of these drugs in future.

EGFR TKIs can sometimes produce remarkable and surprisingly rapid tumour shrinkage and they have the potential to alter tumour biology and the rate of tumour progression. Simply defining a percentage response rate in phase II trials is a sub-optimal approach to EGFR TKI development, and randomised trials with end points such as time to progression, QOL, survival are essential. Wherever possible, trials should be strengthened by the study of pharmacodynmaics with a search for altered tumour biology (proliferation, apoptosis, metabolism). Studies to date have relied upon tumour or skin biopsies, but while these have sometimes been used to guide subsequent trial design, they have not resulted in the identification of a validated, predictive marker for antitumour efficacy. Molecular imaging of pharmacodynamic effects and visualisation of target inhibition is a promising area of research that holds longer-term promise.

We urgently need a more comprehensive understanding of the role of EGFR in human cancer. We must acknowledge that EGFR receptor expression in a tumour does not prove that its function is important for tumour growth, nor that inhibition will automatically result in cell death or therapeutic effect. While the process of clinically validating drug targets is notoriously difficult, EGFR TKIs have relatively specific mechanisms of action, and advances in pharmacodynamics, pharmacogenomics, and genomics/proteomics must be applied in clinical settings to help us realise the full potential of these agents.
